# Aging Forward: Building a Stronger Nation for Older Americans

**DOI:** 10.1093/geroni/igaf122.618

**Published:** 2025-12-31

**Authors:** Samsun Naher

**Affiliations:** University of Washington, Seattle, Washington, United States

## Abstract

Older adults make invaluable contributions to society, not only through their cultural, economic, social, and political impact during their careers but also through caregiving, mentorship, and volunteerism after retirement. However, as they age, they face significant challenges that require thoughtful policy interventions. As a health economics and health services researcher, I am honored to be a part of the Health and Aging Policy Fellowship (HAPF) program. Through this fellowship, I have the opportunity to work with the Administration for Community Living (ACL) on the Interagency Coordinating Committee (ICC) on Healthy Aging and Age-Friendly Communities, which seeks to develop policy solutions that promote healthy aging, improve coordination at all levels of government, enhance service delivery, and eliminate barriers to independence for older adults. In collaboration with the 18 federal agencies and departments included in the ICC, my team is assessing the feasibility of the Strategic Framework for the National Plan on Aging and identifying the most crucial areas for focus at this time. This evaluation is based on feedback collected from multiple sources, including individual and organizational responses via the West Health Input Portal, Gallup polling, listening sessions with older adults in AL, AZ, PA, TX, and Washington, DC, community roundtables, and conference discussions within the aging services network. My role involves synthesizing this feedback, identifying policy gaps, and formulating recommendations to support age-friendly communities. In this presentation, I will share key findings, policy challenges, and proposed solutions to advance a more inclusive and supportive aging policy framework.

